# Nomograms to predict the prognosis in locally advanced oral squamous cell carcinoma after curative resection

**DOI:** 10.1186/s12885-021-08106-x

**Published:** 2021-04-07

**Authors:** Zhiliang Nie, Pengcheng Zhao, Yishan Shang, Bo Sun

**Affiliations:** 1 First Affiliated Hospital of Gannan Medical University, Ganzhou, Jiangxi China; 2 School of Stomatology, Dalian Medical University, Dalian, Liaoning China; 3 Dalian Municipal Women and Children’s Medical Center, Dalian, Liaoning China; 4 The Second Hospital of Dalian Medical University, Dalian, Liaoning China

**Keywords:** Oral squamous cell carcinoma, Nomogram, Cancer management, Prognosis

## Abstract

**Background:**

Oral squamous cell carcinoma (OSCC) is the dominant histologic type of oral cancer. Locally advanced OSCC remains a major therapeutic challenge. Our study aimed to develop and validate nomograms predicting survival prognosis in patients with locally advanced oral squamous cell carcinoma (OSCC) after curative resection.

**Methods:**

A total of 269 consecutive patients with primary OSCC who received curative resection between September 2007 and March 2020 were retrospectively enrolled in our study. Patients were randomly assigned to the training cohort (*n* = 201) or the validation cohort (*n* = 68). Multivariate Cox regression analyses were conducted to determine independent prognostic factors for overall survival (OS) and cancer specific survival (CSS) in the training set, which were used to develop nomogram models estimating 3-, and 5-year OS and CSS. We also evaluated the nomograms using concordance indices (c-index), calibration curves, and decision curve analyses (DCA), and compared those with the AJCC 8th staging system. The results were externally validated in the validation cohort.

**Results:**

Age, Kaplan-Feinstein (KFI) index, pT, the number of positive nodes and systemic inflammatory index (SII) were significant prognostic predictors for OS and CSS. The OS nomogram had c-index values of 0.712 in the training set and 0.697 in the validation set, while the CSS nomogram exhibited c-index values of 0.709 in the training set and 0.675 in the validation set. These data were superior to those of AJCC 8th staging system, suggesting high discriminative ability of the nomograms. Calibration curves exhibited good agreement between observed and predicted survival. DCA curves indicated the nomograms were with potential clinical usefulness. These results were validated in the validation set.

**Conclusions:**

The novel nomograms incorporating clinically available characteristics for OS and CSS prediction were developed in the locally advanced OSCC patients after curative surgery. Validation revealed good discrimination and calibration, indicating the clinical utility of the nomograms in the individualized prognosis prediction of locally advanced OSCC after curative surgery.

## Background

Oral cavity cancer is one of the most common malignancies worldwide, with an estimated incidence of 355,000 new cases per year [1]. Oral squamous cell carcinoma (OSCC), the dominant histologic type of oral cancer, accounts for 95% of oral tumors [2]. The overall age standardized incidence rate is 21 per 100,000 in male and 17 per 100,000 in female [3]. Despite the spreading of multimodal treatment approaches, the prognosis of OSCC, especially locally advanced OSCC, have not improved significantly for the past 30 years [4–6]. Locally advanced OSCC remains a major therapeutic challenge. A better understanding of the prognostic factors is necessary for appropriate risk stratification of patients, optimization of therapeutic approaches and individualization of patient care.

The staging of OSCC based on American Joint Committee on Cancer (AJCC) TNM system has been used for several years to estimate OSCC patients’ survival in clinical practice [7]. However, the traditional TNM staging system only considers several clinical pathological features. In addition to these, the prognosis of OSCC related to a series of factors includes the other clinical pathological features for example, tumor site, tumor grade, and presence of lymphovascular invasion, as well as the patient specific characteristics such as age, smoking and comorbidities [8]. Hence, the consideration of a set of prognostic relevant clinical-pathologic factors could offer more accurate prognostic information.

Various reports have shed light on the probable prognostic significance of certain biomarkers in the setting of OSCC, of which serum biomarkers are of potential clinical utility due to their feasibility and accessibility. Multiple serum biomarkers including lymphocyte count, neutrophil-lymphocyte ratio (NLR), and platelet–lymphocyte ratio (PLR) have been proposed and validated as significant prognosticators in a broad spectrum of cancer [9–12]. Recently, the systemic immune-inflammation index (SII) combining neutrophil, lymphocyte and platelet, has been reported to provide prognostic information in several malignancies. Diao P et al. [13] found that preoperative SII could serve as a powerful prognostic predictor in patients with primary OSCC.

Prognostic models integrating a set of clinical attributes offer greater precision in clinical outcome prediction. Nomograms are statistical tools to visualize complex models that use a set of clinical characteristics for prediction of individual patient’s outcome [14]. Nowadays, nomograms have been widely used as a user-friendly tool to evaluate the prognosis of various cancers [15–17]. What’s more, the recurrence and staging of prostate cancers via nomograms have been included into the NCCN clinical guidelines [18]. However, nomograms for predicting the prognosis of locally advanced OSCC is scarce.

The present study was a retrospective analysis of a cohort of patients with locally advanced OSCC in an academic tertiary care center. The aim of our study was to determine the prognostic significance of different clinical-pathologic factors, and establish the first nomograms using the most relevant prognostic factors to estimate the probabilities of overall survival (OS), and cancer specific survival (CSS) in patients with locally advanced OSCC for better risk stratification and clinical decision-making.

## Methods

### Study subjects

From September 2007 to March 2020, a total of 497 patients with OSCC underwent the curative resection at an university hospital were retrospectively recruited. All patients were histopathologically confirmed of locally advanced (stage III or IV non-metastatic) OSCC. Of these, we excluded 228 patients: 55 had recurrent or metastasized cancer, 29 had other concomitant primary cancer, 25 had other concomitant primary cancer, 40 had preoperative chemotherapy/radiotherapy or contradictions of surgery, 29 had incomplete medical records and 50 were lost to follow up. The remaining 269 patients were enrolled in our study. The primary tumor resection was performed per our institutional guidelines. Following the surgery, the pathological TNM classification was established using the AJCC 8th edition. Postoperative chemotherapy and/or radiotherapy were performed selectively based on our institutional guidelines.

Two thirds of these patients were randomly assigned to the training set (*n* = 201) to establish the predictive models and the remaining patients (*n* = 68) were assigned to the validation set to evaluate the performance of the models.

### Data collection

Patients’ data were collected from electronic records including age at diagnosis, gender, comorbidity, smoking status, preoperative blood tests, TNM stage, tumor grade, presence of perineural invasion, depth of tumor invasion, number of positive nodes, lymphovascular invasion, primary tumor site, extracapsular extension, bone invasion, and safety margins. All identifiable information stored was strictly followed per the hospital’s guidance. Cigarette smokers were defined as individuals who reported having smoked more than 100 cigarettes during their life time and/or smoked every day for at least 1 year. The pathological TNM classification of all tumors was established per the AJCC Staging Manual (2010). Patient comorbidity was assessed by using the Kaplan-Feinstein index (KFI) [19].

The whole blood samples for neutrophil, monocyte and platelet counts were harvested within 3 days before surgery. Systemic inflammatory index (SII), was calculated from preoperative counts of peripheral blood platelets (P), neutrophils (N) and lymphocytes (L) per the following equation: SII = P × N/L. NLR and PLR were defined as follows: NLR = N/L, PLR = P/L. SPSS software was used to investigate the cut-off values of SII, NLR and PLR for OS. The results revealed that the optimal cut-off values were of 535.5 for SII, 2.8 for NLR and 162.5 for PLR.

After discharge, patients were followed-up every 3 months for the first three years, every 6 months until 5 years and annually thereafter. OS was defined as the time interval from the date of surgery to the end of the study or death. CSS was defined as the time elapsed between the date of surgery and the death attributed to OSCC, or the end of the study.

All the procedures involving human participants were in accordance with the ethical standards of the institutional and/or national research committee and with the 1964 Helsinki Declaration and its later amendments or comparable ethical standards. The study has been approved by the ethics committee of The Second Hospital of Dalian Medical University. Informed consent was obtained from all patients.

### Statistical analysis

Continuous variables were compared by Student’s t test or Mann-Whitney U test, while categorical variables were compared by Chi-square test or Fisher’s exact test. OS and CSS were estimated by Kaplan-Meier method and the statistical differences in survival compared by log-rank tests. COX proportional hazards regression models were used to identify the independent prognostic predictors for OS and CSS. Subsequently, these significant predictors in the training set were used to establish nomogram models via the *rms* package in R software. We evaluated the predictive performance by the concordance index (c-index) values and calibration with 1000 bootstrap resampling. The *rcorrcens* command was used to acquire c-index values and the *calibrate* command was to obtain the calibration plots. The c-index was calculated to quantify the predictive accuracy of the nomograms. Calibration plots were generated to check the consistency between the predicted and observed probabilities [20]. In the external validation, the total points of each patient were generated based on the established nomograms. Thereafter, the Cox regression analyses were carried out by using the patients’ total points as a factor. Additionally, c-index values and calibration curves were derived in the validation set. Decision curve analysis (DCA) was employed to examine the clinical net benefit of a predictive model by the *rmda* package in R software [21]. Finally, we compared the performance of the nomograms with AJCC 8th edition TNM staging system by using the c-index values and DCA methods.

All statistical analyses were analyzed using SPSS 22.0 software (SPSS, Inc., Chicago, IL, USA) and R software version 3.5.2 (https://www.r-project.org)with R packages cmprsk, rmda, rms, and survival packages. A two-tailed *P* value < 0.05 was considered statistically significant.

## Results

### Clinicopathological characteristics of the study cohort

A total of 269 OSCC patients (204 male and 65 female), with a median age of 62 years (range, 21–85 years) were enrolled in this study. The tumors were most frequently located on the mobile tongue and floor of the mouth. 67.9% of the patients were well or moderately differentiated. Cervical lymph node dissection was performed in 266 patients. Regarding the treatment, 113 patients received radiotherapy exclusively after surgery, 100 patients underwent concomitant radiotherapy and chemotherapy after surgery, and 51 patients just had surgical resection without adjuvant radiotherapy. Table 1 presented patient demographic and tumor variables of the study cohort. In total, 201 patients were assigned into the training set, while 68 patients were assigned into the validation set. The two groups exhibited similar demographic and tumor parameters (*P* < 0.05).

**Table 1:** Clinicopathological Characteristics of the study cohort

Characteristics	Training cohort(***n*** = 201)	Validation cohort(***n*** = 68)	***P*** Value
Gender			0.639
Female	50 (24.9)	15 (22.1)	
Male	151 (75.1)	53 (77.9)	
Age	62.7 ± 11.6	62.8 ± 11.9	0.956
KFI			0.257
< 2	125 (62.19)	37 (54.41)	
≥ 2	76 (37.81)	31 (45.59)	
Smoking			0.679
No	121 (60.2)	39 (57.35)	
Yes	80 (39.8)	29 (42.65)	
pT			0.359
T1	41 (20.4)	12 (17.65)	
T2	63 (31.34)	21 (30.88)	
T3	54 (26.87)	15 (22.06)	
T4	43 (21.39)	20 (29.41)	
pN			0.259
N0	31 (15.42)	9 (13.24)	
N1	53 (26.37)	14 (20.59)	
N2	110 (54.73)	40 (58.82)	
N3	7 (3.48)	5 (7.35)	
AJCC Stage			0.544
III	61 (30.3)	18 (26.5)	
IV	140 (69.7)	50 (73.5)	
Perineural invasion			0.931
No	120 (59.7)	41 (60.3)	
Yes	81 (40.3)	27 (39.7)	
Tumor differentiation			0.640
Well differentiated	87 (43.3)	25 (36.8)	
Moderately differentiated	51 (25.4)	19 (27.9)	
Poorly differentiated	63 (31.3)	24 (35.3)	
Depth of tumor invasion			0.268
< 20 mm	127 (63.2)	48 (70.6)	
≥ 20 mm	74 (36.8)	20 (29.4)	
Number of Positive Nodes			0.648
0	84 (41.79)	31 (45.6)	
1–2	58 (28.86)	21 (30.9)	
3–4	34 (16.92)	9 (13.2)	
≥ 5	25 (12.44)	7 (10.3)	
Lymphovascular invasion (%)			0.189
No	41 (20.4)	9 (13.24)	
Yes	160 (79.6)	59 (86.76)	
Primary Tumor Site			0.570
Tongue	69 (34.33)	21 (30.88)	
Floor of mouth	60 (29.85)	25 (36.76)	
Other	72 (35.82)	22 (32.35)	
Extracapsular extension			0.537
No	158 (78.61)	51 (75)	
Yes	43 (21.39)	17 (25)	
Bone invasion			0.366
No	119 (59.2)	36 (52.94)	
Yes	82 (40.8)	32 (47.06)	
Safety Margins			0.408
< 5 mm	45 (22.39)	12 (17.65)	
≥ 5 mm	156 (77.61)	56 (82.35)	
SII			0.580
< 535.5	120 (59.7)	38 (55.88)	
≥ 535.5	81 (40.3)	30 (44.12)	
NLR			0.480
< 2.8	125 (62.19)	39 (57.35)	
≥ 2.8	76 (37.81)	29 (42.65)	
PLR			0.761
< 162.5	114 (56.72)	40 (58.82)	
≥ 162.5	87 (43.28)	28 (41.18)	

*Abbreviations*: *AJCC* American Joint Committee on Cancer, *KFI* Kaplan-Feinstein index, *NLR* neutrophil-lymphocyte ratio, *PLR* platelet–lymphocyte ratio, *SII* systematic immune-inflammation index

### Survival analyses

The median follow-up period was 55 months (ranging from 2 to 95 months). At the end of follow-up, a total of 115 patients died, with 74 patients dying of cancer-related causes and 41 patients dying of other causes.

In the training set, the OS rates at 3- and 5-year were 66.4% (95%CI: 63.5–69.3%) and 55.6% (95%CI: 49.5–61.7%), respectively, while the CSS rates at 3- and 5-year were 78.3 and 66.2% respectively. In the validation set, the OS rates at 3- and 5-year were 63.0 and 55.4%, respectively, while the CSS rates at 3- and 5-year were 70.2 and 61.6%, respectively.

### OS

The results of the univariate and multivariate models for OS are provided in Table 2. Age, KFI index, pT, pN, AJCC stage, the number of positive nodes, NLR, PLR and SII were significant predictors of OS in the univariate analysis. In the multivariate analysis results showed that, age (*P* < 0.001), KFI index (*P* = 0.003), pT (*P* < 0.001), the number of positive nodes (*P* = 0.009) and SII (*P* < 0.001) remained to be significant prognosticators.

**Table 2:** Univariate and multivariate analyses of OS in locally advanced OSCC patients

Characteristics	Univariate analysis	*P* Value	Multivariate analysis	*P* Value
	HR(95%CI)		HR(95%CI)	
Gender				
Female	Ref			
Male	1.034 (0.908–1.179)	0.612		
Age	1.024 (1.014–1.035)	**< 0.001**	1.023 (1.012–1.033)	**< 0.001**
KFI				
< 2	Ref		Ref	
≥ 2	1.389 (1.087–1.776)	**0.009**	1.458 (1.137–1.871)	**0.003**
Smoking				
No	Ref			
Yes	1.284 (0.904–1.824)	0.163		
pT		**< 0.001**		**< 0.001**
T1	Ref		Ref	
T2	2.317 (1.424–3.771)	0.001	2.128 (1.303–3.477)	0.003
T3	2.869 (1.79–4.598)	0	3.157 (1.945–5.124)	0
T4	4.317 (2.728–6.831)	0	4.316 (2.674–6.966)	0
pN		**.073**		.065
N0	Ref		Ref	
N1	0.641 (0.445–0.924)	0.017	1.031 (0.701–1.517)	0.876
N2	0.825 (0.618–1.101)	0.192	1.358 (0.99–1.864)	0.058
N3	1.078 (0.656–1.773)	0.766	1.685 (1.01–2.811)	0.046
AJCC Stage				
III	Ref		Ref	
IV	1.526 (1.863–1.251)	**< 0.001**	1.358 (0.99–1.864)	0.058
Perineural invasion				
No	Ref			
Yes	1.428 (0.778–2.618)	0.250		
Tumor differentation		0.224		
Well differentiated	Ref			
Moderately differentiated	1.385 (0.856–2.241)	0.184		
Poorly differentiated	2.581 (0.452–14.724)	0.286		
Depth of tumor invasion				
< 20 mm	Ref			
≥ 20 mm	1.385 (0.856–2.241)	0.184		
Number of Positive Nodes		**0.002**		0.009
0	Ref		Ref	
1–2	1.483 (1.112–1.979)	0.007	1.357 (1.013–1.819)	0.041
3–4	1.725 (1.238–2.402)	0.001	1.549 (1.106–2.171)	0.011
≥ 5	1.782 (1.203–2.64)	0.004	1.786 (1.202–2.653)	0.004
Lymphovascular invasion (%)				
No	Ref			
Yes	2.581 (0.452–14.724)	0.286		
Primary Tumor Site		0.245		
Tongue	Ref			
Floor of mouth	2.625 (0.726–9.486)	0.141		
Other	1.306 (0.939–1.817)	0.113		
Extracapsular extension				
No	Ref			
Yes	2.377 (0.583–9.689)	0.227		
Bone invasion				
No	Ref			
Yes	1.244 (0.887–1.743)	0.206		
Safety Margins				
< 5 mm	Ref			
≥ 5 mm	1.397 (0.766–2.547)	0.276		
SII				
< 535.5	Ref		Ref	
≥ 535.5	1.724 (1.354–2.194)	< 0.001	1.599 (1.25–2.047)	< 0.001
NLR				
< 2.8	Ref		Ref	
≥ 2.8	1.774 (1.09–2.885)	0.021	2.243 (0.751–6.704)	0.148
PLR				
< 162.5	Ref		Ref	
≥ 162.5	2.472 (1.127–5.424)	0.024	1.204 (0.854–1.699)	0.289

*Abbreviations*: *AJCC* American Joint Committee on Cancer, *KFI* Kaplan-Feinstein index, *NLR* neutrophil-lymphocyte ratio, *PLR* platelet–lymphocyte ratio, *SII* systematic immune-inflammation index

### CSS

In the univariate analysis, age, KFI index, pT, pN, AJCC stage, the number of positive nodes, safety margins, SII, NLR and PLR had a statistically significant impact on CSS. The results of multivariate model demonstrated the following variables as potential independent risk factors of CSS: age (*P* < 0.001), KFI index (*P* = 0.003), pT (*P* = 0.003), the number of positive nodes (*P* = 0.008) and SII (*P* < 0.001) (Table 3).

**Table 3:** Univariate and multivariate analyses of CSS in locally advanced OSCC patients

Characteristics	Univariate analysis		Multivariate analysis	
	SHR(95%CI)	*P* Value	SHR(95%CI)	*P* Value
Age	1.372 (1.221–1.541)	**0**	1.05 (1.029–1.071)	**0**
Gender				
Female	Ref			
Male	1.034 (0.908–1.179)	0.612		
KFI				
< 2	Ref		Ref	
≥ 2	1.389 (1.087–1.776)	**0.009**	1.706 (1.109–2.624)	0.015
Smoking				
No	Ref			
Yes	1.284 (0.904–1.824)	0.163		
pT		**0**		**0.004**
T1	Ref		Ref	
T2	2.317 (1.424–3.771)	**0.001**	3.122 (1.252–7.782)	**0.015**
T3	2.869 (1.79–4.598)	**0**	5.073 (2.077–12.392)	**0**
T4	4.317 (2.728–6.831)	**0**	4.428 (1.762–11.129)	**0.002**
pN		**0.073**		.625
N0	Ref		Ref	
N1	0.641 (0.445–0.924)	0.017	0.871 (0.451–1.68)	0.68
N2	0.825 (0.618–1.101)	0.192	1.127 (0.648–1.96)	0.671
N3	1.078 (0.656–1.773)	0.766	1.575 (0.626–3.965)	0.335
AJCC Stage				
III	Ref		Ref	
IV	1.728 (1.413–2.113)	**0**	0.750 (0.513–1.095)	0.136
Perineural invasion				
No	Ref			
Yes	1.225 (0.844–1.777)	0.285		
Tumor differentation		0.235		
Well differentiated	Ref			
Moderately differentiated	1.774 (0.627–5.016)	0.28		
Poorly differentiated	2.252 (0.61–8.315)	0.223		
Depth of tumor invasion				
< 20 mm	Ref			
≥ 20 mm	2.298 (0.537–9.834)	0.262		
Number of Positive Nodes		**.009**		0.009
0	Ref		Ref	
1–2	1.564 (1.076–2.272)	0.019	1.432 (1.003–2.044)	0.048
3–4	2.079 (1.307–3.308)	0.002	1.679 (1.097–2.569)	0.017
≥ 5	2.487 (1.572–3.935)	0.0001	2.413 (1.349–4.318)	0.003
Lymphovascular invasion (%)				
No	Ref			
Yes	2.672 (0.639–11.172)	0.178		
Primary Tumor Site				
Tongue	Ref			
Floor of mouth	1.815 (0.593–5.55)	0.296		
Other	1.865 (0.775–4.483)	0.164		
Extracapsular extension				
No	Ref			
Yes	1.866 (0.775–4.494)	0.164		
Bone invasion				
No	Ref			
Yes	1.689 (0.789–3.614)	0.177		
Safety Margins				
< 5 mm	Ref			
≥ 5 mm	1.699 (0.772–3.74)	0.188		
SII				
< 535.5	Ref		Ref	
≥ 535.5	2.732 (1.149–6.498)	0.023	2.214 (1.289–3.805)	0.004
NLR				
< 2.8	Ref		Ref	
≥ 2.8	1.164 (1.022–1.326)	**0.022**	1.502 (0.729–3.096)	0.27
PLR				
< 162.5	Ref		Ref	
≥ 162.5	1.861 (1.048–3.304)	**0.034**	2.246 (0.732–6.885)	0.157

*Abbreviations*: *AJCC* American Joint Committee on Cancer, *KFI* Kaplan-Feinstein index, *NLR* neutrophil-lymphocyte ratio, *PLR* platelet–lymphocyte ratio, *SII* systematic immune-inflammation index

### Nomograms construction

**Fig. 1:**
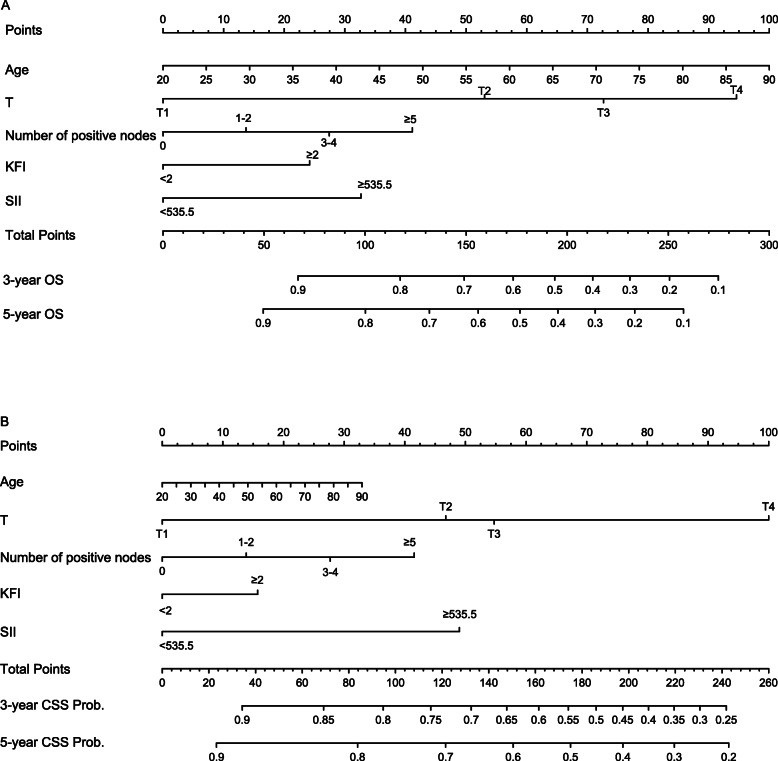
Nomograms for prognosis prediction in locally advanced OSCC patients. **a** 3-, and 5-year OS; **b** 3-, and 5-year CSS. Draw a straight line up to the point axis to determine the points assigned for each covariate. Sum the points and locate the total points on the bottom scale to determine the possibilities of 3- and 5-year OS and CSS in locally advanced OSCC patients. The higher total points indicate the lower expected survival. OS, overall survival; CSS, cancer specific survival

Based on the results of COX regression analyses, we constructed prognostic nomograms for 3-year and 5-year OS and CSS (Fig. 1). The point of each factor can be determined by drawing the vertical line from the variable to the point axis. By summing up the total score and locating it on the total point scale, we can get the estimated survival probability at each time point.

### Nomogram validation

**Fig. 2:**
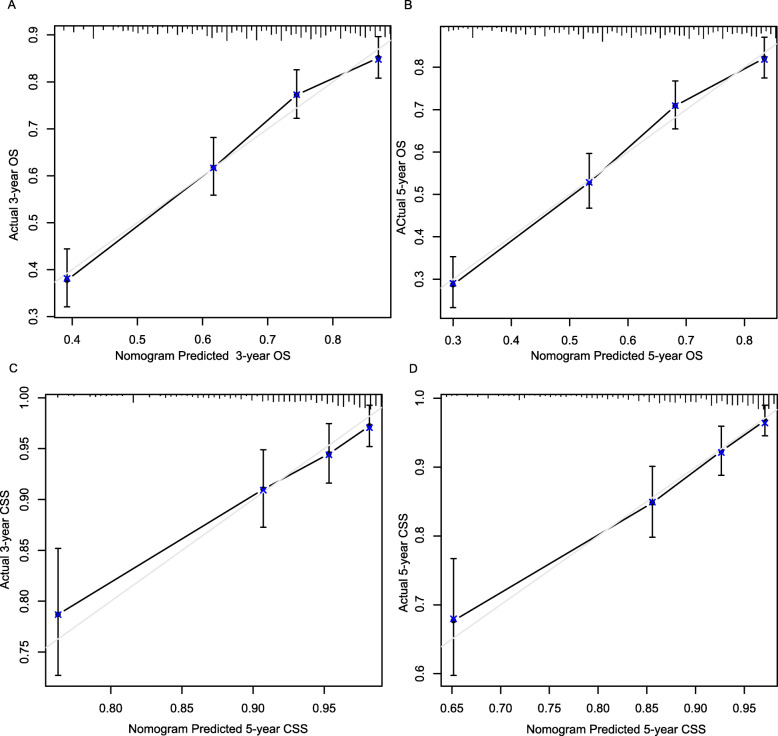
Calibration curves of nomograms in locally advanced OSCC patients in the validation cohort. **a** 3-year OS; **b** 5-year OS; **c** 3-year CSS; **d**5-year CSS. OS, overall survival; CSS, cancer specific survival

Nomogram validation was assessed using the c-index values and calibration curves. The c-index values showed that the established nomograms had good discriminative abilities with 0.712 for OS and 0.709 for CSS. Figure 2 showed the calibration plots for the nomogram models of OS and CSS in the training set, in which nomograms predicated OS and CSS probabilities were well matched with the actual probabilities.

**Fig. 3:**
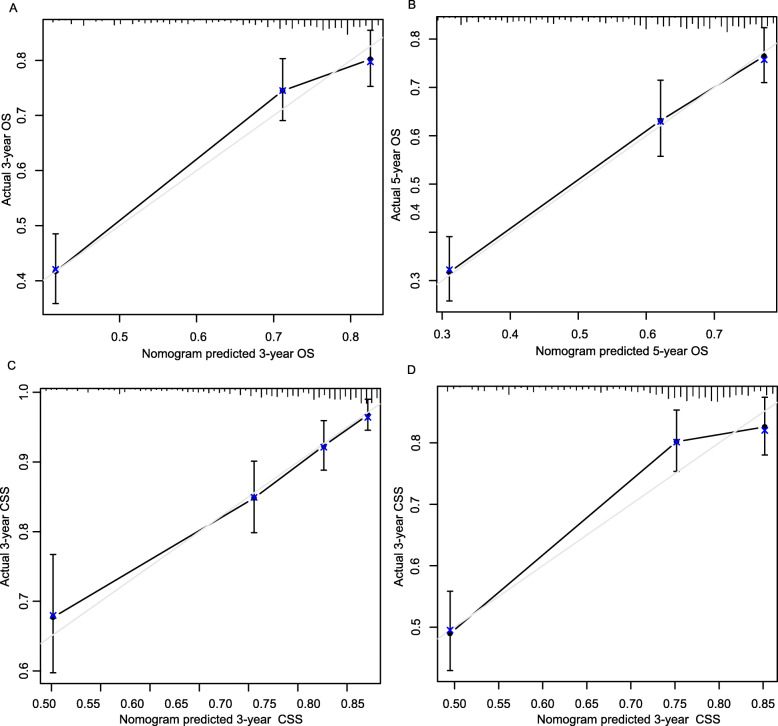
Calibration curves of nomograms in locally advanced OSCC patients in the training cohort. **a** 3-year OS; **b** 5-year OS; **c** 3-year CSS; **d**5-year CSS. OS, overall survival; CSS, cancer specific survival

When subjected to the external validation, the nomograms also discriminated to a good extent with c index values of 0.697 for OS and 0.675 for CSS. Calibration plots (Fig. 3) indicated the good consistency between nomograms predicted and actual probabilities in validation set, which suggested the good accuracy of the established nomograms.

### Survival analyses according to the risk stratification based on the nomograms

**Fig. 4:**
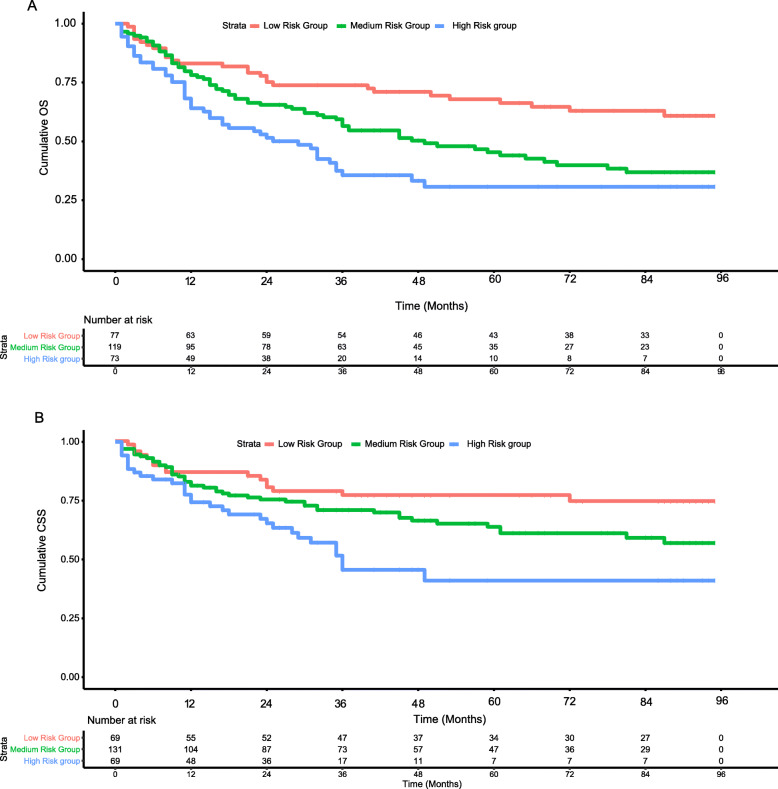
Kaplan-Meier survival curves and risk group stratification within all the patients based on the quartiles of nomograms predictions. **a** OS; **b** CSS. OS, overall survival; CSS, cancer specific survival

The total points of each patient were generated from the established nomograms. All the patients were evenly divided into three subgroups per the total points. With regards for OS, the three groups were low risk group (≤145), medium risk group (145–190), and high risk group (>190). For CSS, the three groups were low risk group (≤102), medium risk group (102–141), and high risk group (>141). As shown in Fig. 4, patients in the high risk group had distinctly lower OS and CSS survival probabilities (*P* < 0.001).

### Comparison of the nomogram with the AJCC staging

**Fig. 5:**
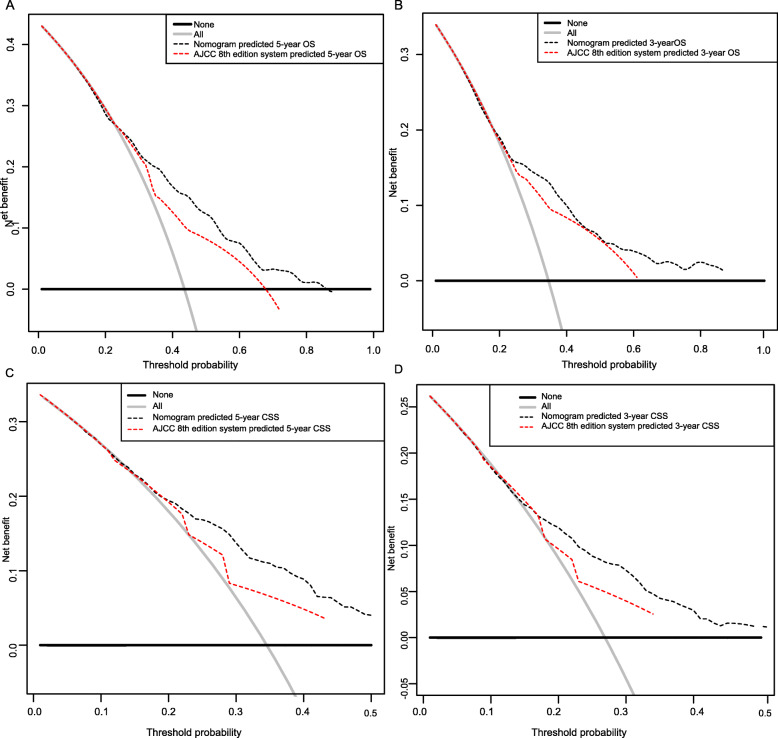
Decision curve analysis for the nomogram model and AJCC 8th staging system in the validation cohort. (A) 3-year OS; (B) 5-year OS; (C) 3-year CSS; (D)5-year CSS. AJCC: American Joint Committee on Cancer; OS, overall survival; CSS, cancer specific survival

**Fig. 6:**
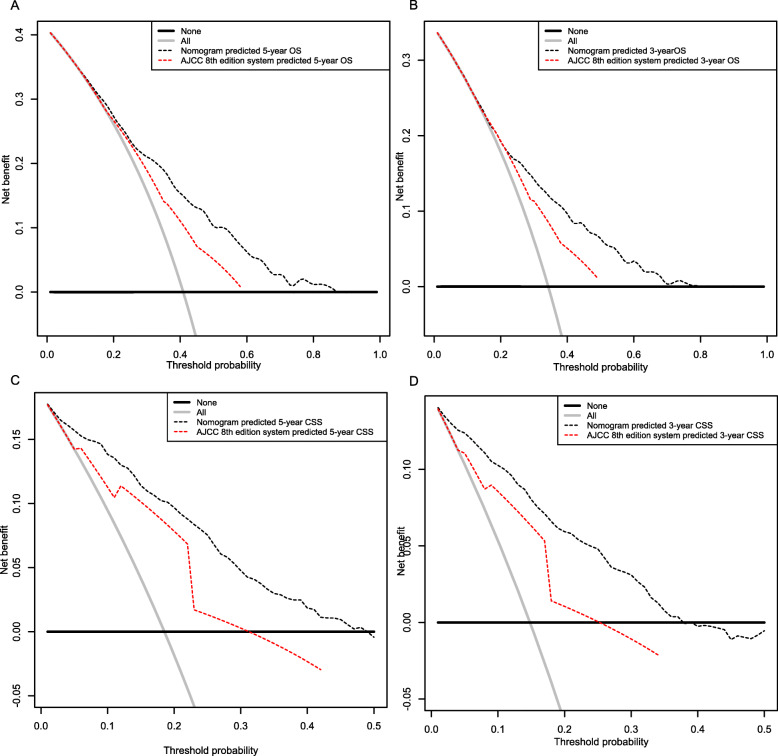
Decision curve analysis for the nomogram model and AJCC 8th staging system in the training cohort. **a** 3-year OS; **b** 5-year OS; **c** 3-year CSS; **d**5-year CSS. AJCC: American Joint Committee on Cancer; OS, overall survival; CSS, cancer specific survival

Compared to the AJCC staging system, our nomograms had statistically higher c-indices for OS and CSS prediction in locally advanced OSCC patients, which were summarized in Table 4. DCA has been proposed as a method to assess the clinical validity of the prediction models. The DCA plots demonstrated the established nomogram models were associated with improved clinical net benefits over the AJCC stages with wider ranges of threshold probabilities in both training (Fig. 5) and validation sets (Fig. 6).

**Table 4:** Comparison of the nomograms with the AJCC staging

	Nomogram score	8th AJCC stage
Training Cohort		
OS	0.712 (0.683–0.741)	0.567 (0.563–0.571)
CSS	0.709 (0.691–0.727)	0.611 (0.593–0.629)
Validation Cohort		
OS	0.697 (0.664–0.73)	0.582 (0.549–0.615)
CSS	0.675 (0.651–0.699)	0.598 (0.574–0.622)

*Abbreviations*: *AJCC* American Joint Committee on Cancer, *CSS* cancer specific survival, *OS* overall survival

## Discussion

Nomograms enable visualize the prognostic strength of various relevant factors in a single model which allow them to have more accurate survival prediction than conventional TNM staging system or an individual molecular biomarker. Nomograms have been widespread used in the prognosis prediction in clinical oncology. Compared with other cancers, nomograms have been sparingly studied for head and neck tumors. For OSCC, several studies have reported on the development of nomograms to predict the survival [22–25]. However, to our knowledge, there was no study specifically for the locally advanced OSCC patients. The present study was the first attempt to investigate the usage of nomograms for survival prediction of locally advanced OSCC.

Our nomograms were constructed based on the COX proportional hazards regression analyses in the training cohort of 201 locally advanced OSCC patients after curative surgery. In the multivariate analyses, we found that advanced age, KFI, pT, the number of positive nodes and SII were significant prognosticators for OS and CSS. Based on these significant prognosticators, we developed the nomograms for OS and CSS. The nomograms showed good discrimination abilities with c-index values of 0.712 for OS and 0.709 for CSS. Calibration curves demonstrated satisfactory agreement between the nomograms and actual survival. Moreover, the nomograms exhibited the net clinical benefit using DCA. We also externally validated the nomograms performance in a validation cohort of 68 patients. External validation also supported the satisfactory accuracy and calibration of our nomograms. Besides, the performance of nomograms was, in turn, validated by Kaplan-Meier curves which showed distinct prognosis in three subgroups sorting by the total points of the nomograms.

The significant prognosticators incorporated in our nomograms were clinically feasible and economical, especially including the novel preoperative systemic inflammation-immune biomarker SII. Notably, accumulating evidence demonstrated that inflammatory cells including neutrophils, platelets, monocytes and lymphocytes carry out a robust role in contributing to proliferation and survival of malignant cells, angiogenesis and metastasis [26]. Many reports also have revealed the significant prognostic values of preoperative systemic inflammation-immune biomarkers, for example, NLR, PLR and LMR, in various types of cancers [9–12]. Recently, SII based on neutrophils, lymphocytes and platelets, has been proved as a novel integrated biomarker and exhibited prognostic value in several tumors including advanced pancreatic cancer [27], cervical cancer [28], gastric cancer [29] and colorectal cancer [30]. The study published in 2018 [13] reported for the first time that increased preoperative SII level was associated with poor outcome and could be served as an independent prognostic predictor for OSCC. Elevated SII probably resulted from neutrophilia, thrombocythemia and lymphopenia. Solid tumor-related neutrophilia, after excluding obvious reasons such as infections, bone marrow metastasis and the usage of corticosteroid, may arise from hematopoietic colony-stimulating factors and inflammatory cytokines triggered by tumors including granulocyte colony-stimulating factor and others [31, 32]. Neutrophils could facilitate tumor growth by the secretion of various chemokines and cytokines, as well as actively recruiting other tumor-supporting cells to the tumor microenvironment [33]. What’s more, tumor associated neutrophils play a critical role in the metastasis process by inhibiting the activity of natural killer cells and enhancing the extravasation of tumor cells, mainly through secreting various matrix metalloproteinases to degrade and modify the extracellular matrix [34]. Thrombocythemia usually promote tumor progression and metastasis. Studies showed that abnormally elevated platelet count over 3.5 × 10^11^/L probably increased cancer risk by 3% in one year of observation [35, 36]. A meta-analysis reported platelet quantity could be a potential prognostic marker in pancreatic cancer [37]. Tumors firstly activate platelets through tissue factors-containing microparticles (MPs). The platelet MPs can express signals and communicate with a variety of cells to induce angiogenesis [38, 39]. Also platelets or platelet activation can directly interact with cancer cells, synergistically promotes TGF-β and NF-kB pathways in cancer which in turn triggers the epithelial mesenchymal transition of cancer cells to facilitate tumor metastasis [36]. Lymphopenia has been frequently observed in patients with advanced cancers and shown as a powerful prognostic factor in advanced solid tumors including renal cell carcinoma, colorectal, lung cancer and breast cancer [40–43]. Lymphocytes as major immune cells exert a fundamental role in cell-mediated immunologic destruction of cancer cells, although different subtypes of lymphocytes vary in their functional roles against cancer [44, 45]. Thus, lymphopenia could be considered as indicative of impaired immune surveillance and contribute to the favorable tumor microenvironment for tumor metastasis. In our study, multivariate analyses revealed that SII was a powerful prognosticator of OS and CSS in advanced OSCC.

Concerning clinical-pathologic factors, the most important prognosticators were age, comorbidity, depth of invasion (DOI), extranodal extension (ENE), number of positive nodes, perineural invasion (PNI) and tumor grade. Advanced age and greater comorbidity have been reported by various studies on the upper aerodigestive tract tumors, as elderly patients or patients in poor general health are more vulnerable to disease progression and not eligible for invasive therapies. Consistent with previous findings, our data also confirmed advanced age and greater comorbidity as the independently clinical prognostic factors in advanced OSCC patients. Tumor grade wasn’t identified as a prognostic factor in our data, which probably can be explained by the homogeneity of the study population in terms of patients and tumor profiles.

DOI has been advocated to be associated with tumor metastasis and worse survival outcomes, and included in the AJCC 8th T staging classification. In our study, pT classification was independently associated with worse prognosis. Lymph nodal involvement has been a well-established prognostic factor in head and neck cancers. The AJCC 8th staging has included lymph nodal site (ipsilateral and contralateral), size, presence of ENE in the nodal staging category. The negative impact of ENE has been fully incorporated in the AJCC 8th N staging system, where it leads to upstaging nodal positive OSCC, whatever size, number, or laterality of the positive node(s) [7]. However, the number of lymph node is probably overestimated in the system. AJCC 8th N staging classified patients with more than one lymph node as N2, without further stratification for the increasing number of positive lymph nodes. Several clinical studies have observed the prognostic significance of number of positive lymph nodes in OSCC, albeit with different cut-off values [46]. Roberts et al. [47] reported that the number of positive lymph nodes model (0, 1, 2–4 and ≥ 5) performed better than AJCC 7th edition N staging model in head and neck cancers. Moreover, a recent publication by Rajappa et al. [48] revealed that the number of positive lymph nodes (0, 1, 2, > 2) outperformed AJCC 8th nodal staging system in the prediction of OS and disease free survival in oral cancer. Subramaniam et al. [49] categorized the number of positive lymph nodes as 0, 1–2, 3–4 and ≥ 5 and exhibited it was superior to LNR and log odds of positive lymph nodes in the prediction of OS and DFS in 643 OSCC patients. In our study, we adopted the categorization system proposed by Subramaniam et al. [49]. We also observed the inverse relationship between the number of positive lymph nodes and patients’ survival, and confirmed its prognostic significance for OS and CSS in advanced OSCC patients.

Based on the independent prognosticators discussed above, we built the first nomograms predicting OS and CSS in locally advanced OSCC patients and internal and external validations showed our models with relatively high c-indices and well-fitted calibration curves. Currently, AJCC 8th staging system is the widely used system for assessment of prognosis in locally advanced OSCC patients. We performed comparative analysis between our developed nomograms and AJCC staging system. Our nomograms outperformed the AJCC 8th staging system for OS and CSS prediction in locally advanced OSCC patients, with statistically higher c-indices. Additionally, in the DCA analyses, the nomograms exhibited to be more beneficial over AJCC 8th staging system in the prognosis prediction of OS and CSS. These data demonstrated that our nomograms had better performance with clinical utility in prognosis prediction.

The present study had two main limitations. Firstly, our study was a retrospective study so that the selection bias was inevitable. Secondly, the patients enrolled were from a single institution, which may not represent the entire locally advanced OSCC patients. Notwithstanding these limitations, our study built the first nomograms predicting OS and CSS in locally advanced OSCC patients. More importantly, robust internal and external validation demonstrated sufficient discriminatory power and accurate calibration in our proposed nomograms. Additionally, the main advantages of the present study were that all the included prognosticators were feasible and accessible in daily clinical practice.

## Conclusions

In conclusion, we constructed and validated nomograms based on clinically available characteristics for predicting 3- and 5- year OS and CSS in patients with locally advanced OSCC. The novel nomograms displayed relatively good performance with potential clinical utility, which would aid the individualized risk stratifying the patients and contribute to the individualized disease management.

## Data Availability

The data used in the current study are available from the corresponding author upon reasonable request.

## Abbreviations

AJCC: American Joint Committee on Cancer
CSS: Cancer specific survival
DOI: Depth of invasion
ENE: Extranodal extension
HR: Hazard ratio
NLR: Neutrophil-lymphocyte ratio
OSCC: Oral squamous cell carcinoma
OS: Overall survival
PLR: and platelet–lymphocyte ratio
PNI: Perineural invasion
SII: Systemic immune-inflammation index

## References

[1] Bray F, Ferlay J, Soerjomataram I, Siegel RL, Torre LA, Jemal A (2018). Global cancer statistics 2018: GLOBOCAN estimates of incidence and mortality worldwide for 36 cancers in 185 countries. CA Cancer J Clin.

[2] Chinn SB, Myers JN (2015). Oral cavity carcinoma: current management, controversies, and future directions. J Clin Oncol.

[3] Ferlay J, Soerjomataram I, Dikshit R, Eser S, Mathers C, Rebelo M, et al. Cancer incidence and mortality worldwide: sources, methods and major patterns in GLOBOCAN 2012. Int J Cancer. 2015;136(5):E359–86. 10.1002/ijc.29210. 10.1002/ijc.29210 25220842

[4] Thompson-Harvey A, Yetukuri M, Hansen AR, Simpson MC, Adjei Boakye E, Varvares MA, et al. Rising incidence of late-stage head and neck cancer in the United States. Cancer. 2020;126(5):1090–101. 10.1002/cncr.32583. 10.1002/cncr.32583 31722124

[5] Maekawa A, Ishihara R, Iwatsubo T, Nakagawa K, Ohmori M, Iwagami H, et al. High incidence of head and neck cancers after endoscopic resection for esophageal cancer in younger patients. J Gastroenterol. 2020;55(4):401–7. 10.1007/s00535-019-01653-y. 10.1007/s00535-019-01653-y 31813008

[6] Ghantous Y, Yaffi V, Abu-Elnaaj I. [Oral cavity cancer: epidemiology and early diagnosis]. Refuat Hapeh Vehashinayim (1993) 2015; 32(3): 55–63, 71. 26548152

[7] Moeckelmann N, Ebrahimi A, Tou YK, Gupta R, Low TH(H), Ashford B, et al. Prognostic implications of the 8th edition American joint committee on Cancer (AJCC) staging system in oral cavity squamous cell carcinoma. Oral Oncol. 2018;85:82–6. 10.1016/j.oraloncology.2018.08.013. 10.1016/j.oraloncology.2018.08.013 30220324

[8] Vincent N, Dassonville O, Chamorey E, Poissonnet G, Pierre CS, Nao EEM, et al. Clinical and histological prognostic factors in locally advanced oral cavity cancers treated with primary surgery. Eur Ann Otorhinolaryngol Head Neck Dis. 2012;129(6):291–6. 10.1016/j.anorl.2012.01.004. 10.1016/j.anorl.2012.01.004 23149218

[9] Perisanidis C, Kornek G, Poschl PW (2013). High neutrophil-to-lymphocyte ratio is an independent marker of poor disease-specific survival in patients with oral cancer. Med Oncol.

[10] Wang Y, Wang P, Andrukhov O, Wang T, Song S, Yan C, et al. Meta-analysis of the prognostic value of the neutrophil-to-lymphocyte ratio in oral squamous cell carcinoma. J Oral Pathol Med. 2018;47(4):353–8. 10.1111/jop.12688. 10.1111/jop.12688 29406591

[11] Tangthongkum M, Tiyanuchit S, Kirtsreesakul V, Supanimitjaroenporn P, Sinkitjaroenchai W (2017). Platelet to lymphocyte ratio and red cell distribution width as prognostic factors for survival and recurrence in patients with oral cancer. Eur Arch Otorhinolaryngol.

[12] Zhang Y, Zheng L, Quan L, du L (2019). Prognostic role of platelet-to-lymphocyte ratio in oral cancer: a meta-analysis. J Oral Pathol Med.

[13] Diao P, Wu Y, Li J, Zhang W, Huang R, Zhou C, et al. Preoperative systemic immune-inflammation index predicts prognosis of patients with oral squamous cell carcinoma after curative resection. J Transl Med. 2018;16(1):365. 10.1186/s12967-018-1742-x. 10.1186/s12967-018-1742-x PMC6299596 30563540

[14] Balachandran VP, Gonen M, Smith JJ, DeMatteo RP (2015). Nomograms in oncology: more than meets the eye. Lancet Oncol.

[15] Wang XH, Long LH, Cui Y, Jia AY, Zhu XG, Wang HZ, et al. MRI-based radiomics model for preoperative prediction of 5-year survival in patients with hepatocellular carcinoma. Br J Cancer. 2020;122(7):978–85. 10.1038/s41416-019-0706-0. 10.1038/s41416-019-0706-0 PMC7109104 31937925

[16] Peintinger F (2011). Clinical use of nomograms for breast cancer. J Surg Oncol.

[17] Thurtle DR, Jenkins V, Pharoah PD, Gnanapragasam VJ (2019). Understanding of prognosis in non-metastatic prostate cancer: a randomised comparative study of clinician estimates measured against the PREDICT prostate prognostic model. Br J Cancer.

[18] Mohler JL, Antonarakis ES, Armstrong AJ, D’Amico AV, Davis BJ, Dorff T, et al. Prostate Cancer, version 2.2019, NCCN clinical practice guidelines in oncology. J Natl Compr Cancer Netw. 2019;17(5):479–505. 10.6004/jnccn.2019.0023. 10.6004/jnccn.2019.0023 31085757

[19] Kaplan MH, Feinstein AR (1974). The importance of classifying initial co-morbidity in evaluating the outcome of diabetes mellitus. J Chronic Dis.

[20] Xu J, Shi KQ, Chen BC, Huang ZP, Lu FY, Zhou MT (2017). A nomogram based on preoperative inflammatory markers predicting the overall survival of pancreatic ductal adenocarcinoma. J Gastroenterol Hepatol.

[21] Huang J, Liu FC, Li L, Zhou WP, Jiang BG, Pan ZY (2020). Nomograms to predict the long-time prognosis in patients with alpha-fetoprotein negative hepatocellular carcinoma following radical resection. Cancer Med.

[22] Mattavelli D, Lombardi D, Missale F, Calza S, Battocchio S, Paderno A, et al. Prognostic Nomograms in Oral squamous cell carcinoma: the negative impact of low neutrophil to lymphocyte ratio. Front Oncol. 2019;9:339. 10.3389/fonc.2019.00339. 10.3389/fonc.2019.00339 PMC6503119 31114760

[23] Wang F, Zhang H, Wen J, Zhou J, Liu Y, Cheng B, et al. Nomograms forecasting long-term overall and cancer-specific survival of patients with oral squamous cell carcinoma. Cancer Med. 2018;7(4):943–52. 10.1002/cam4.1216. 10.1002/cam4.1216 PMC5911576 29512294

[24] Kao HK, Lofstrand J, Loh CY (2018). Nomogram based on albumin and neutrophil-to-lymphocyte ratio for predicting the prognosis of patients with oral cavity squamous cell carcinoma. Sci Rep.

[25] Montero PH, Yu C, Palmer FL, Patel PD, Ganly I, Shah JP, et al. Nomograms for preoperative prediction of prognosis in patients with oral cavity squamous cell carcinoma. Cancer. 2014;120(2):214–21. 10.1002/cncr.28407. 10.1002/cncr.28407 24399417

[26] Colotta F, Allavena P, Sica A, Garlanda C, Mantovani A. Cancer-related inflammation, the seventh hallmark of cancer: links to genetic instability. Carcinogenesis. 2009;30(7):1073–81. 10.1093/carcin/bgp127. 10.1093/carcin/bgp127 19468060

[27] Zhang K, Hua YQ, Wang D, Chen LY, Wu CJ, Chen Z, et al. Systemic immune-inflammation index predicts prognosis of patients with advanced pancreatic cancer. J Transl Med. 2019;17(1):30. 10.1186/s12967-019-1782-x. 10.1186/s12967-019-1782-x PMC6339361 30658662

[28] Huang H, Liu Q, Zhu L, Zhang Y, Lu X, Wu Y, et al. Prognostic value of preoperative systemic immune-inflammation index in patients with cervical Cancer. Sci Rep. 2019;9(1):3284. 10.1038/s41598-019-39150-0. 10.1038/s41598-019-39150-0 PMC6397230 30824727

[29] Shi H, Jiang Y, Cao H, Zhu H, Chen B, Ji W (2018). Nomogram based on systemic immune-inflammation index to predict overall survival in gastric Cancer patients. Dis Markers.

[30] Chen JH, Zhai ET, Yuan YJ, Wu KM, Xu JB, Peng JJ, et al. Systemic immune-inflammation index for predicting prognosis of colorectal cancer. World J Gastroenterol. 2017;23(34):6261–72. 10.3748/wjg.v23.i34.6261. 10.3748/wjg.v23.i34.6261 PMC5603492 28974892

[31] Wilcox RA (2010). Cancer-associated myeloproliferation: old association, new therapeutic target. Mayo Clin Proc.

[32] Banerjee R, Roxin G, Eliasziw M, Joseph K, MacLean A, Buie WD, et al. The prognostic significance of pretreatment leukocytosis in patients with anal cancer treated with radical chemoradiotherapy or radiotherapy. Dis Colon Rectum. 2013;56(9):1036–42. 10.1097/DCR.0b013e31829ab0d4. 10.1097/DCR.0b013e31829ab0d4 23929012

[33] Shaul ME, Fridlender ZG (2018). Cancer-related circulating and tumor-associated neutrophils - subtypes, sources and function. FEBS J.

[34] Granot Z, Fridlender ZG (2015). Plasticity beyond cancer cells and the "immunosuppressive switch". Cancer Res.

[35] Ankus E, Price SJ, Ukoumunne OC, Hamilton W, Bailey SER (2018). Cancer incidence in patients with a high normal platelet count: a cohort study using primary care data. Fam Pract.

[36] Huong PT, Nguyen LT, Nguyen XB, et al. The Role of Platelets in the Tumor-Microenvironment and the Drug Resistance of Cancer Cells. Cancers (Basel) 2019;11(2) doi: 10.3390/cancers11020240, The Role of Platelets in the Tumor-Microenvironment and the Drug Resistance of Cancer Cells, 11, 2. 10.3390/cancers11020240 PMC6406993 30791448

[37] Chen S, Na N, Jian Z (2018). Pretreatment platelet count as a prognostic factor in patients with pancreatic cancer: a systematic review and meta-analysis. Onco Targets Ther.

[38] Caine GJ, Lip GY, Blann AD (2004). Platelet-derived VEGF, Flt-1, angiopoietin-1 and P-selectin in breast and prostate cancer: further evidence for a role of platelets in tumour angiogenesis. Ann Med.

[39] Chater C, Bauters A, Beugnet C, M’Ba L, Rogosnitzky M, Zerbib P (2018). Intraplatelet vascular endothelial growth factor and platelet-derived growth factor: new biomarkers in Carcinoembryonic antigen-negative colorectal Cancer?. Gastrointest Tumors.

[40] Ray-Coquard I, Cropet C, Van Glabbeke M (2009). Lymphopenia as a prognostic factor for overall survival in advanced carcinomas, sarcomas, and lymphomas. Cancer Res.

[41] Crocenzi T, Cottam B, Newell P, Wolf RF, Hansen PD, Hammill C, et al. A hypofractionated radiation regimen avoids the lymphopenia associated with neoadjuvant chemoradiation therapy of borderline resectable and locally advanced pancreatic adenocarcinoma. J Immunother Cancer. 2016;4(1):45. 10.1186/s40425-016-0149-6. 10.1186/s40425-016-0149-6 PMC4986363 27532020

[42] Ohtani H (2007). Focus on TILs: prognostic significance of tumor infiltrating lymphocytes in human colorectal cancer. Cancer Immun.

[43] Mehrazin R, Uzzo RG, Kutikov A, et al. Lymphopenia is an independent predictor of inferior outcome in papillary renal cell carcinoma. Urol Oncol 2015; 33(9): 388 e19–25. doi: 10.1016/j.urolonc.2014.06.004 10.1016/j.urolonc.2014.06.004 PMC4289664 25027688

[44] Gajewski TF, Schreiber H, Fu YX (2013). Innate and adaptive immune cells in the tumor microenvironment. Nat Immunol.

[45] Bindea G, Mlecnik B, Tosolini M, Kirilovsky A, Waldner M, Obenauf AC, et al. Spatiotemporal dynamics of intratumoral immune cells reveal the immune landscape in human cancer. Immunity. 2013;39(4):782–95. 10.1016/j.immuni.2013.10.003. 10.1016/j.immuni.2013.10.003 24138885

[46] Ho AS, Kim S, Tighiouart M, Gudino C, Mita A, Scher KS, et al. Metastatic lymph node burden and survival in Oral cavity Cancer. J Clin Oncol. 2017;35(31):3601–9. 10.1200/JCO.2016.71.1176. 10.1200/JCO.2016.71.1176 PMC5791830 28880746

[47] Roberts TJ, Colevas AD, Hara W, Holsinger FC, Oakley-Girvan I, Divi V (2016). Number of positive nodes is superior to the lymph node ratio and American joint committee on Cancer N staging for the prognosis of surgically treated head and neck squamous cell carcinomas. Cancer.

[48] Rajappa SK, Maheshwari U, Jaipuria J, Singh AK, Goyal S, Batra U, et al. Number of positive nodes - current relevance in determining prognosis of oral cavity cancer after the recent AJCC staging update. Oral Oncol. 2019;90:1–5. 10.1016/j.oraloncology.2019.01.001. 10.1016/j.oraloncology.2019.01.001 30846166

[49] Subramaniam N, Balasubramanian D, Kumar N, Murthy S, Vijayan SN, Nambiar A, et al. Lymph node staging systems in oral squamous cell carcinoma: a comparative analysis. Oral Oncol. 2019;97:92–8. 10.1016/j.oraloncology.2019.08.002. 10.1016/j.oraloncology.2019.08.002 31465931

